# Nutritional support and mortality in critically ill adults - a subset analysis of the calories trial

**DOI:** 10.1186/2197-425X-3-S1-A284

**Published:** 2015-10-01

**Authors:** T Kilner, E Bidgood, S Benham-Mirando, R Krol, D Brealey

**Affiliations:** University College London Hospital, Critical Care, London, United Kingdom

## Introduction

University College London Hospital (UCLH) was one of the largest contributors to the CALORIES trial; a pragmatic, multi-centre, randomised control trial examining the effectiveness of early nutritional support in critically ill patients through a comparison of parenteral and enteral nutrition. The trial found no significant difference in 30-day mortality associated with the route of calorie delivery [1]. Both groups had a similar calorie intake and neither group attained their calorie target. We sought to determine whether our institution's nutritional support practices were concordant with the CALORIES trial findings, and whether a relationship between calorie 'dose' and patient outcomes could be delineated [1].

## Objectives

At UCLH, we sought to determine whether there was an association between:

1) Type of nutritional support and meeting a calorie target

2) Type of nutritional support and 30, and 90 day mortality

3) Meeting a calorie target and 30, and 90 day mortality

## Methods

Data from 138 CALORIES participants' trial records at UCLH were collected. Type of nutritional support was defined as parenteral or enteral. Meeting a calorie target was defined as a trial participant receiving an average of 25 kcals/kg/day during the first five days in critical care [1]. Data were analysed using Stata 11.0. Logistic regression and ANCOVA analyses were conducted.

## Results

67 (48.6%) participants were randomised to receive enteral nutrition and 71 (51.5%) parenteral. A mean of 12.6 (SD 7.2) kcals/kg/day/patient was delivered enterally and 17.4 (SD 5.8) kcals/kg/day/patient parenterally. Nine participants (6.5%) met their calorie target. There was strong evidence of an association between a parenteral nutrition strategy and achieving 50% of target calorie intake (p < 0.001). After adjusting for covariates, the parenteral group achieved a significantly higher mean proportion of their target calorie intake compared with the enteral group (p < 0.001). However, no statistically significant associations were found between:

i) type of nutritional support and meeting target calorie requirements;

ii) meeting the calorie target and 30 or 90 day mortality; and

iii) type of nutritional strategy and 30 or 90 day mortality.

## Conclusions

In contrast to the aggregated results of CALORIES, parenterally fed, critically ill patients at UCLH achieved a significantly greater proportion of their target calorie intake than those enterally fed. However, there was no relationship between calories delivered and outcome. In concordance with the CALORIES findings, our analysis showed the route of nutrition was not associated with mortality. Our results suggest that further work needs to be done to establish the relationship between calorie intake and outcome.

## Grant Acknowledgment

No funding received.
